# Use of High Density Single Nucleotide Polymorphism (SNP) Arrays to Assess Genetic Diversity and Population Structure of Dairy Cattle in Smallholder Dairy Systems: The Case of Girinka Programme in Rwanda

**DOI:** 10.3389/fgene.2018.00438

**Published:** 2018-10-10

**Authors:** Mizeck G. G. Chagunda, Fidalis D. N. Mujibi, Theogene Dusingizimana, Olivier Kamana, Evans Cheruiyot, Okeyo A. Mwai

**Affiliations:** ^1^Animal Breeding and Husbandry in the Tropics and Subtropics, University of Hohenheim, Stuttgart, Germany; ^2^Usomi Limited, Nairobi, Kenya; ^3^The Nelson Mandela African Institution of Science and Technology, Arusha, Tanzania; ^4^College of Agriculture, Animal Sciences and Veterinary Medicine, University of Rwanda, Kigali, Rwanda; ^5^International Livestock Research Institute, Nairobi, Kenya

**Keywords:** genetic diversity, population structure, dairy cattle, smallholder, SNP arrays

## Abstract

In most smallholder dairy programmes, farmers are not fully benefitting from the genetic potential of their dairy cows. This is in part due to the mismatch between the available genotypes and the environment, including management, in which the animals perform. With sparse performance and pedigree records in smallholder dairy farms, the true degree of baseline genetic variability and breed composition is not known and hence rendering any genetic improvement initiative difficult to implement. Using the Girinka programme of Rwanda as an exemplar, the current study was aimed at better understanding the genetic diversity and population structure of dairy cattle in the smallholder dairy farm set up. Further, the association between farmer self-reported cow genotypes and genetically determined genotypes was investigated. The average heterozygosity estimates were highest (0.38 ± 0.13) for Rwandan dairy cattle and lowest for Gir and N’Dama (0.18 ± 0.19 and 0.25 ± 0.20, respectively). Systematic characterization of the genetic variation and diversity available may inform the formulation of sustainable improvement strategies such as targeting and matching the genotype of cows to productivity goals and farmer profile and hence reducing the negative impact of genotype by environment interaction.

## Introduction

Smallholder dairying has the potential to drive people out of poverty, provide sustainable livelihoods and enhance household food and nutritional security. In different countries in Sub-Saharan Africa, a variety of dairy development initiatives are being implemented either by national governments or Non-Governmental Organisations (NGOs) (Chagunda et al., 2016). An example of such initiatives is the “One Cow per Poor Family Programme” in Rwanda. This programme, which is locally known as “Girinka,” is a country-wide initiative to provide poor households with dairy cattle. This target is to especially provide cattle in areas where there is currently low cattle population. The Girinka programme was launched in 2006 with the overall objective of increasing agricultural productivity through application of cow manure in crop field and also through increased dairy production. This in turn would drive improvements in human nutrition, household income and reduced poverty. According to the Rwandan Ministry of Agriculture, a total of 249,000 cows of different breeds had been distributed by June 2016. In addition to cattle of known breeds such as Ankole, Jersey, Ayrshire, and Holstein Friesian, cross-bred cows of different grades have also been distributed to farmers. Some of the animals were sourced from within the country while the majority of the animals were imported from countries such as Kenya, Uganda, Tanzania, South Africa, and Netherlands. Such an importation strategy not only changes the genotypic frequency at population level, but also increases the genetic diversity of the base population. The Girinka programme is a classic example of the different variants of smallholder dairy programme development in Sub-Saharan Africa. Key to any future improvement initiatives is the use of breed composition information to target and match genetics to productivity goals. The challenge, though is that with sparse performance and pedigree records in smallholder dairy farms, the true degree of baseline genetic variability and breed composition is not known and hence difficult to implement any meaningful genetic improvement initiative. The objective of the current study was to better understand the genetic diversity and population structure of dairy cattle under the Girinka programme through use of high density single nucleotide polymorphism (SNP) arrays. This approach has the potential to clearly inform the formulation of sustainable improvement strategies.

## Materials and Methods

### Ethics Statement

#### Ethical Approval

All procedures performed in the study involving human participants and the protocol for animal hair sample collection were reviewed and approved by the Ethics Committee of the University of Rwanda’s Research and Postgraduate Studies (RPGS) Unit and the National Institute of Statics Rwanda (NISR) based on the guidelines provided by the Rwanda National Ethics Committee and in accordance with the 1964 Helsinki declaration and its later amendments or comparable ethical standards. Animal handling was done by knowledgeable personnel to ensure maximum comfort and minimal injury at all stages of the research.

### Farmer Survey and Animal Samples

This study was conducted as a survey that combined social economic data, data on indictor traits for cow productivity, biological data in terms of animal hair samples. All the numerators were properly trained to conduct the survey and all standard biosecurity and institutional safety procedures were adhered to under the supervision of the expert from the University of Rwanda. A total of 1564 smallholder dairy farmers from the South and North provinces of Rwanda were interviewed. The respondents were beneficiaries of the Girinka programme. Socio-economic and productivity data that were collected included information on gender issues, production systems, access to relevant dairy production inputs such as fodder, water, labour, and animal health services. Animal hair samples were collected from the tail switch, taking care to avoid faecal contamination, following a protocol provided by the International Livestock Research Institute (ILRI). A total of 2717 cows were sampled from smallholder dairy farms consisting of 1492 samples from the North province and 12245 samples from the South province. Due to budget limitations a total of 150 random samples were selected from each of the provinces and shipped for genotyping. Samples were heat treated at 70°C for 2 h in preparation for shipping and genetic analysis. Of the 300 submitted samples, genotyping results were obtained from 299 samples. The rest of the samples have been safely stored in a biorepository at ILRI for future use. Results from the socio-economic survey are beyond the scope of the current paper.

### Reference Dataset

A panel of genotypes from commercial international taurine dairy breeds was used as a reference for breed composition assignment. These included Friesian (*n* = 28 samples), Holstein (*n* = 63), Norwegian Red (*n* = 17), Jersey (*n* = 36), and Guernsey (*n* = 21) breeds. To capture genetic signatures representative of African cattle, an African taurine breed (N’Dama (*n* = 24)) and two indicine breeds, the East African Shorthorn Zebu (EASZ) (*n* = 50) and Gir (*n* = 30) were also included in the analysis.

### Genotyping and Quality Control

Samples were genotyped at Geneseek (Neogen Corporation, Nebraska, United States) using the Geneseek Genomic Profiler (GGP) High Density (HD) SNP array consisting of 150,000 SNPs, while SNPs for the reference breeds had been genotyped with the Illumina HD Bovine (777K SNPs) array. The SNPs in GGP array were optimised for use in dairy cattle having the most informative SNPs from Illumina Bovine 50k and 770k chips and additional variants known to have a large effect on disease susceptibility and performance. Genotype data quality control and cheques were carried out using PLINK v 1.9 (Purcell et al., 2007) and included removal of SNPs with less than 90% call rate, less than 5% minor allele frequency (MAF) and samples with more than 10% missing genotypes. Additional removal of SNPs not mapped to any chromosome left a total of 120,591 SNPs for analysis. Of the 299 animals, 12 failed the above outlined quality cheques and were removed from the analysis. Total genotyping rate in remaining samples was 0.991. The 120,591 SNPs used in the analysis covered 2516.25 Mb with an average distance of 22.67 kb between adjacent SNPs. The mean chromosomal length ranged between 42.8 Mb on BTA 25 and 158.86 Mb on BTA 1. The mean length of adjacent SNPs per chromosome ranged between 18.67 and 23.89 kb on BTA 14 and BTA 29, respectively. The linkage disequilibrium (LD) across the genome averaged 0.41. Private alleles, defined as variants which are segregating in only one population when evaluating multiple populations, were identified using a custom script in R. A total of 143 private variants, most (132) of which originated from the Rwanda cattle population were detected.

### Minor Allele Frequency, Inbreeding and Heterozygosity Estimates

Minor allele frequencies (MAF) were estimated using PLINK. The distribution of MAF in each subpopulation (i.e., European taurine, African taurine, Indicine breeds and Tanzanian crossbred cattle) was represented as the proportion of all the SNPs used in the analysis and subsequently grouped into five classes as follows: [0.0,0.1], [0.1,0.2], [0.2,0.3], [0.3,0.4], and [0.4,0.5]. The results were plotted for comparison between subpopulations using R (R Core Team, 2016). The observed heterozygosity estimates for each population were calculated from observed genotype frequencies obtained from PLINK (Purcell et al., 2007) using the programme Hierfstat. Inbreeding coefficient estimates were also calculated using the Hierfstat package (Goudet, 2005) in R (R Core Team, 2016). To obtain confidence intervals, 100,000 permutations after pruning such that markers were in approximate linkage equilibrium were performed. Pruning was carried out in PLINK programme using the –indep-pairwise (50 5 0.3) option. The pruning proceeded by calculating LD for 50 marker sliding windows, with a new window obtained by shifting 5 markers along the length of the chromosome. Marker pruning was carried out when LD between a pair of markers was either 0.3 or above. Consequently, 33,208 markers were removed leaving a total of 87,383 markers that were used for the inbreeding analysis. Negative *F*_IS_-values was set to zero because such inbreeding coefficient estimates reflects sampling error (Purcell et al., 2007).

### Admixture and Principal Component Analysis

Principal component analysis (PCA) was used to describe the genetic structure of the crossbred cattle population using PLINK (Purcell et al., 2007) by way of a variance-standardised relationship matrix for dimension reduction. The PCA results were visualised using the GENESIS package (Buchmann and Hazelhurst, 2014) in R. The unsupervised model-based clustering method implemented by the programme ADMIXTURE v. 1.3.0 (Alexander et al., 2009) was used to estimate the breed composition of individual animals using 111,836 markers. The analysis was undertaken with K (number of distinct breeds) ranging from 2 to 9 to reflect the genetic background of the cattle under study, starting with the basic cross (indicine and taurine cross) until the total number of the populations in the analysis, given the 8 reference breeds. Ten-fold cross-validation (CV = 10) was specified, with the error profile obtained thereafter used to explore the most probable number of clusters (K), as described by Alexander et al. (2009). Graphical display of the admixture output was done using the Genesis package (Buchmann and Hazelhurst, 2014) in R statistical programme (R Core Team, 2016).

### Phylogeny and Pairwise Fst

In order to understand the relationships between the populations, the Euclidean distance between populations was evaluated using dartR package (Gruber et al., 2018) in R. A Neighbour-joining (NJ) relationship tree was then constructed using APE programme (Paradis et al., 2004). Pairwise population differentiation was calculated using Hierfstat. Confidence intervals were obtained after 100,000 permutations.

## Results

Farmer-self reported information showed that the predominant genotype (45%) used for milk production in the Girinka programme was the cross between Holstein-Friesian and Zebu (**Table 1**). Ten percent of the farmers received pure Holstein-Friesian cattle while 6% farmers received Jersey cattle. Other farmers received local Zebu (20%). Quite a substantial proportion (18%) of farmers did not know the genotype of the cow that they received. From the genetic analysis, the majority (87%) of the cows was determined as cross-breeds between exotic dairy breeds such as Holstein Friesian, Jersey and Ayrshire; and local zebu type of animals. The rest were either pure exotic breeds (7%) or local zebu breeds (6%). There was 46.2% agreement and 29.4% disagreement between the farmer-reported genotypes and the genetically determined genotypes. The rest of the animals (24.4%) had their owners reporting that they did not know the genotype at all. The majority of the farmers received the animals as either calves (66%) or growing heifers (24%).

**Table 1:** Farmer self-reported cow genotype and age at which the first animal was received from the Girinka Programme.

	Genotype								Age			
		
Gender of Household Head	*n*	Local zebu	Ankole	Cross bred	Jersey	Holstein Friesian	Don’t know	Other^1^	Calf	Heifer	Pregnant cow	Mature
Male	861	19%	1%	47%	7%	10%	16%	1%	66%	23%	5%	7%
Female	703	20%	1%	43%	4%	10%	21%	1%	66%	24%	5%	4%
Total	1564	20%	1%	45%	6%	10%	18%	1%	66%	24%	5%	6%


*^1^Other breeds included Guernsey, Norwegian Red and other crosses.*

### Genetic Diversity

**FIGURE 1:**
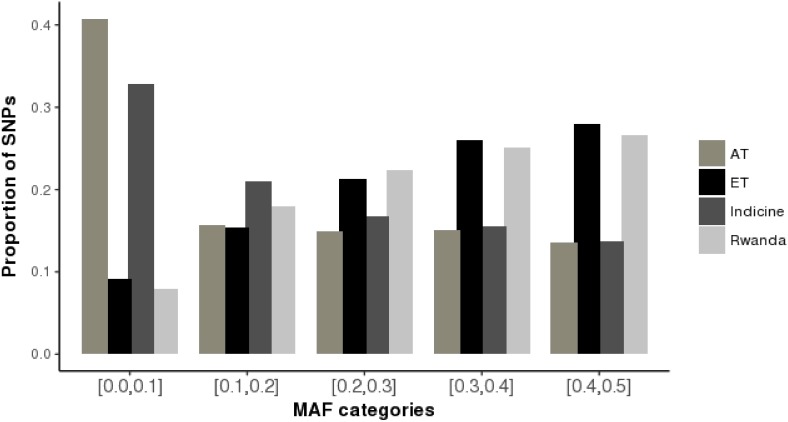
Minor allele frequency distributions for Rwanda cattle and reference breeds. AT, African taurine; ET, 0 European taurine; Indicine, East African Shorthorn Zebu and Gir; Rwanda, Girinka cattle population.

The distributions of average minor allele frequencies for all populations under study (African taurine, Indicine, and Rwandan crossbred cattle) are shown in **Figure 1**. Indicine (EASZ and Gir) and African taurine (N’Dama) breeds had the highest proportion of SNPs with the low MAF category ([0.0,0.1]) compared to European taurine (ET) breeds. The Rwandan crossbred cattle had relatively high proportion of SNPs with high MAF (mostly [0.3,0.4] and [0.4,0.5]). The observed heterozygosity estimates for the study populations are illustrated in **Table 2**. The average heterozygosity estimates were high for the Rwanda cattle (0.38 ± 0.13) and lowest for Gir and N’Dama (0.18 ± 0.19 and 0.25 ± 0.20, respectively). Heterozygosity estimates for European taurine breeds used as references ranged between 0.30 ± 0.19 and 0.37 ± 0.12 for Jersey and Holstein breeds, respectively.

**Table 2:** Average inbreeding coefficient, observed and expected heterozygosity estimates. Values are means ± SD.

Population	Inbreeding coefficient	MAF	Observed heterozygosity (H_o_)	Expected heterozygosity (H_e_)
Rwanda	0.008 ± 0.069	0.29 ± 0.13	0.378 ± 0.129	0.383 ± 0.127
Friesian	0.011 ± 0.225	0.28 ± 0.14	0.355 ± 0.10	0.368 ± 0.144
Holstein	-0.001 ± 0.176	0.29 ± 0.14	0.365 ± 0.155	0.372 ± 0.134
Norwegian Red	0.002 ± 0.255	0.27 ± 0.15	0.352 ± 0.185	0.36 ± 0.154
Guernsey	0.018 ± 0.218	0.24 ± 0.16	0.318 ± 0.185	0.326 ± 0.172
Jersey	0.008 ± 0.216	0.23 ± 0.16	0.304 ± 0.192	0.312 ± 0.174
N’Dama	0.011 ± 0.200	0.18 ± 0.16	0.246 ± 0.204	0.25 ± 0.196
EASZ	0.039 ± 0.167	0.20 ± 0.15	0.261 ± 0.177	0.274 ± 0.175
Gir	0.024 ± 0.222	0.13 ± 0.15	0.176 ± 0.191	0.181 ± 0.185


The study populations showed low detectable levels of inbreeding for both Rwanda cattle and the reference samples (**Table 2**). The values obtained were not significantly different from zero.

**FIGURE 2:**
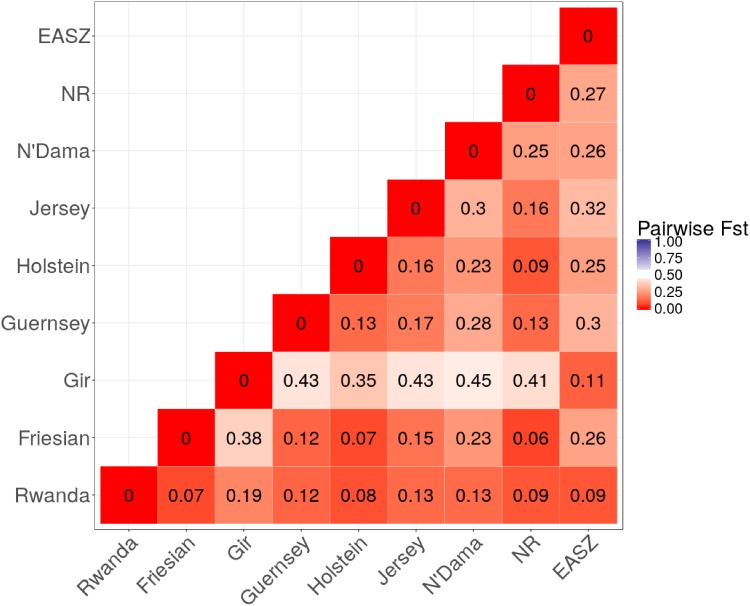
Heat map of pairwise Fst among study populations.

**Figure 2** shows a heatmap of population differentiation for the Rwanda cattle and the reference populations. For Rwanda cattle, the *F*_st_-values were small ranging from 0.07 to 0.19 for Friesian and Gir, respectively. Large differentiation ranging from 0.35 to 0.43 was observed between Gir and Taurine breeds, reflecting the historical divergence between these breeds (Loftus et al., 1994).

### Principal Coordinate Analysis

**FIGURE 3:**
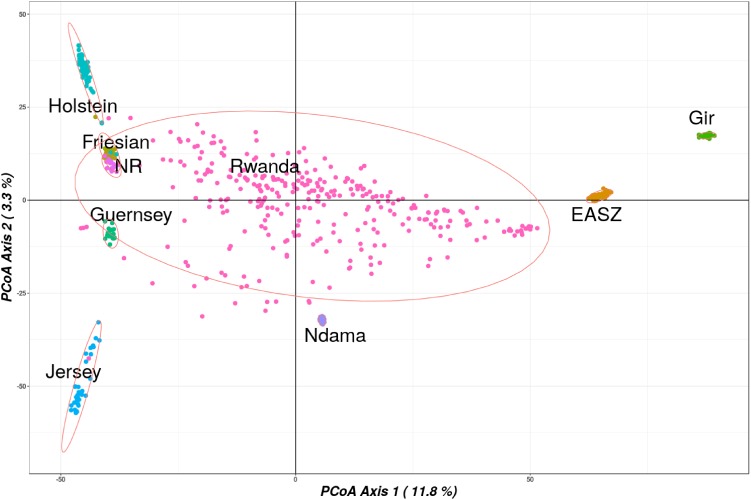
Principle coordinate analysis results showing spatial relationships between the Rwanda and reference populations. Abbreviated reference breeds are Norwegian Red (NR) and East African Shorthorn Zebu (ZB).

The first principal coordinate vector accounted for 12% of the total variation and separated European taurine breeds from non-European breeds as shown in **Figure 3**. The second vector accounted for 3.3% of total variation and separated the African taurine breeds (N’Dama) from the indicine breeds. The Rwandan samples dispersed intermediate between EASZ and the Taurine breeds. A significant number of the Rwandan samples dispersed close to the N’Dama breed, suggesting a significant contribution of the breed in some of the animals in the population.

### Admixture Analysis and Relationship Among the Studied Breeds

**FIGURE 4:**
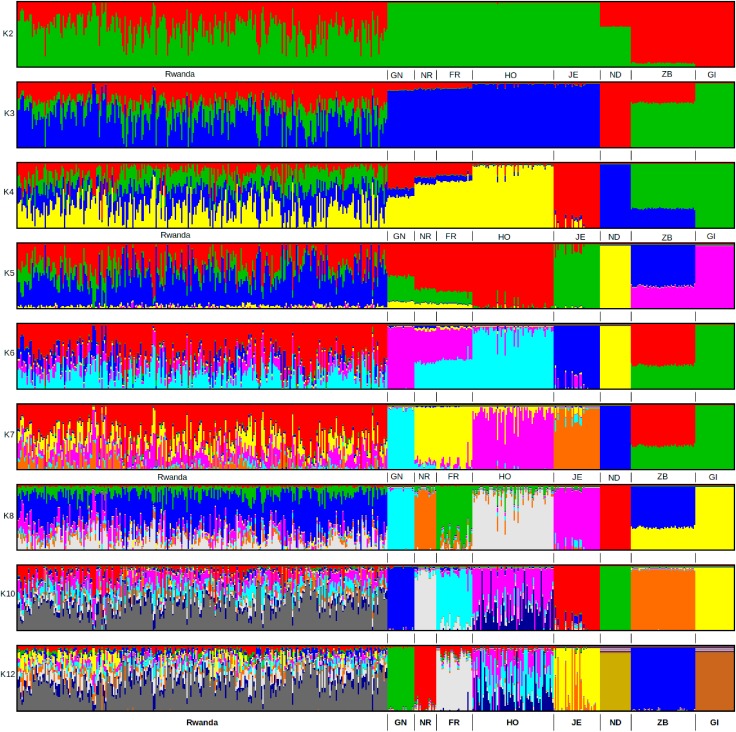
ADMIXTURE bar plots showing breed proportions at assumed ancestries (clusters) *K* = 2 to 12. Reference breeds are labelled as Guernsey (GN), Norwegian Red (NR), Friesian (FR), Holstein (HO), Jersey (JE), N’Dama (ND), East African Shorthorn Zebu (ZB), and Gir (GI).

**FIGURE 5:**
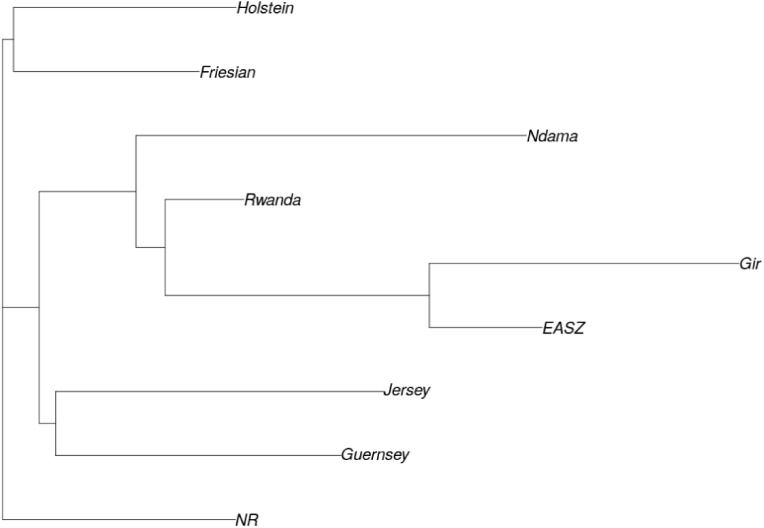
Phylogenic tree showing relationships between study populations. Breeds are labelled as Guernsey (GN), Norwegian Red (NR), Friesian (FR), Holstein (HO), Jersey (JE), N’Dama (ND), Rwanda cattle (RD), East African Shorthorn Zebu (EAZB), and Gir (GI).

ADMIXTURE analysis results are presented in **Figure 4**. Each animal is represented by a vertical line divided into K coloured segments representing the estimated fraction belonging to each cluster. Short vertical lines at the bottom of each horizontal bar delimit individuals of different populations. Reference breeds are labelled as Guernsey (GN), Norwegian Red (NR), Friesian (FR), Holstein (HO), Jersey (JE), N’Dama (ND), East African Shorthorn Zebu (ZB) and Gir (GI). Based on visual inspection of the admixture plot, scrutiny of the separate CV error plots and the PCoA plots, *K* = 8 represented the most appropriate population number for the dataset. Importantly, increasing *K* above 8 did not reveal any detectable population substructure and the breed clusters remained the same. Based on results obtained with *K* = 8, most animals were crosses of Holstein-Friesian breeds which contributed on average 58.3% of the total genes in the crossbred animals. The predicted absolute exotic breed gene content in the crossbred cattle ranged from 12 to 100% (Huson and Bryant, 2006). The phylogenic tree showing the relationships among the studied breeds is presented in **Figure 5**. The phylogeny confirms that the majority of the cows in the Girinka are crosses between the African indicus breeds and the European taurine breeds.

## Discussion

The Girinka programme was introduced by the government of Rwanda as a means of enhancing food and nutritional security for rural poor households. Based on the national poverty assessment, every poor family is mandated to have a dairy cow which provides milk for household nutrition and extra milk is sold to supplement other income streams. Dairy farming lends itself as a pathway out of poverty given its ability to generate a daily household cash flow while keeping the animal alive. However, for the programme to be sustainable, there is need to ensure that farmers access the right animals for their specific production environments. Dairy farmers in the tropics, and specifically in smallholder farms, face many challenges including disease pressure, poor feed availability, high temperatures and generally inappropriate management strategies. A better understanding of the genetic diversity of the population under study is not only important for maximising productivity but also provides a means to evaluate the germplasm supply chains. This would ensure that appropriate animals are sourced for any rural development initiative as well as for any genetic improvement programme. This is vital, not only for enhanced food and nutritional security but also for improved animal welfare.

The results from the current study indicate low genetic diversity in indicine (EASZ and Gir) and African taurine (N’Dama) breeds compared to European taurine (ET) breeds. This result is consistent with the design of the genotyping array used which targets *Bos taurus* breeds, and has low representation of indicine breeds (Bovine HapMap Consortium, 2009). This ascertainment bias causes the disproportionate distribution of MAF among the subpopulations, such that indicine and African breeds had lower diversity measures. The Rwanda population had a relatively large proportion of SNPs with high MAF given their frequent crossbreeding events predominantly with breeds of high European Taurine ancestry. Typically, the study animals are sourced from many smallholder farmers in diverse countries in the region (Hahirwa and Karinganire, 2017). This is because the demand of high quality heifers in East Africa is so high compared to available supply Staal et al. (1996) and Muriuki and Thorpe (2001). There are no large breeders to fill this gap. As such, a few animals are sourced from small herds which are dominated by smallholder farmers (Muriuki and Thorpe, 2001). The high genetic variability observed in the current populations presents an opportunity for implementation of genetic improvement programmes to facilitate adaptation to local production environments which are constantly changing due to continuous environmental perturbations, capacity of farmers to manage the animals and availability of feed resources (Thornton, 2010). The relatively low heterozygosity estimates for indicine and African taurine breeds observed in this study due to poor representation of SNPs for non-European Taurine cattle. It is interesting to note that the Rwanda cattle population had a large proportion of African taurine breed (N’Dama) signature. This represents significant crossbreeding with Ankole cattle, which are Sanga type cattle breed with 50% African taurine and 50% Zebu ancestry. The Rwanda cattle population therefore consists of a unique genepool that can be harnessed to develop a synthetic breed with the best attributes of all cattle breeds in East Africa. This would have the potential to contribute to not only for higher production potential, but also for adaptability to heat and disease stress.

The results also showed minimal differences in inbreeding coefficient estimates between European taurine and the Rwanda population. Given the huge admixture observed for the Rwanda population, this was expected. To accurately assess population structure of the study populations, we chose the PCoA method to assess dissimilarity between populations. The PCoA plot illustrates the wide range of genetic composition and breed contribution. The Rwanda cattle in the Girinka programme are not only highly admixed but also mainly crosses of Holstein Friesian, African taurine (N’Dama) and the East African Zebu. The dispersion pattern observed in this study reflects the practised indiscriminate crossbreeding, where farmer’s continually upgrade their animals to high exotic levels in a bid to increase productivity. ADMIXTURE results agree with the PCoA results and demonstrate the wide range of breed types that constitute the Rwanda Girinka cattle. The dominance of Holstein-Friesian breeds over other cattle breeds reflects the goals for the Girinka programme, in terms of maximising milk yields.

Farmers’ ability to identify the genotype of their animals was limited. This implies that farmers either have poor knowledge of dairy breeds or the animals are not performing as expected. Based on the phenotypic performance of their animals, farmers may not have been convinced that the breed that they were told they would receive is the one they have when it does not perform at the level that the farmers expected. This could be in terms of both underperforming as well as over performing. This mismatch in terms of the breed that the farmers has and what they believe they have also reflect on poor pedigree record keeping and poor access to breed choices. Currently, there are no large farms that would provide large numbers of suitable animals, when needed. A scheme for appropriate sire selection and animal identification ought to be instituted across east Africa. In the meantime, handlers of the Girinka programme need to start instituting a breed composition profiling campaign after they purchase the animals so that they can match animals to specific farmer production systems. Farmers with the capacity to provide the right inputs such as animal feed, proper health management and have access to markets should receive the animals with the highest taurine composition, while those farmers with low capacity to provide inputs, ought to receive animals with a composition consistent with their production system. To ensure that the Girinka programme fulfils its goal, farmer education on dairy best practises and with consideration to cow genetic diversity must precede farmer acquisition of the cattle. This will ensure that farmers are well prepared with regard to the demands of rearing dairy cattle and have the requisite knowledge and inputs. The low dairy productivity reported in different countries in Sub-Saharan Africa reflects the inappropriateness of the breed allocation programmes and also general lack of proper preparatory work done prior to breed allocation.

## Conclusion

This study has demonstrated that a substantial number of farmers in the Girinka programme did not know the real breed of their cow. This would be a major bottleneck in any efforts for breed improvement. The application of high density SNP markers can be used in smallholder production settings to inform decision making and offer insightful options in breed development and distribution among smallholder farmers. Such information is vital in developing future breed sourcing strategies and development efforts among governments and NGOs targeting smallholder farmers. Further, the diversity of breed types used and the wide admixture spread presents the Rwandan dairy population with the opportunity for in-depth studies to identify the appropriate breed types and admixture level for different production systems.

## Data Availability

The data supporting the conclusions of this manuscript has been uploaded to Figshare by the authors at https://doi.org/10.6084/m9.figshare.7046768. Requests for the reference genotypes must be made directly to the owners of this data, as indicated.

## Author Contributions

MC, TD, and OM conceived and designed the study. TD and OK oversaw the data and sample collection. FM and EC conducted the data analysis. MC, FM, TD, OK, EC, and OM wrote the manuscript.

## Conflict of Interest Statement

The authors declare that the research was conducted in the absence of any commercial or financial relationships that could be construed as a potential conflict of interest.
